# A Novel Solubility-Enhanced Rubusoside-Based Micelles for Increased Cancer Therapy

**DOI:** 10.1186/s11671-017-2054-4

**Published:** 2017-04-13

**Authors:** Meiying Zhang, Tongcheng Dai, Nianping Feng

**Affiliations:** 1 Department of Pharmaceutical Sciences, School of Pharmacy, Shanghai University of Traditional Chinese Medicine, 1200 Cailun Road, Pudong New District, Shanghai, 201203 China; 2 Department of Pharmaceutics, School of Pharmacy, Key Laboratory of Smart Drug Delivery, Ministry of Education, Fudan University, Shanghai, 201203 China; 3 State Key Laboratory of Molecular Engineering of Polymers, Fudan University, Shanghai, 200433 China

**Keywords:** Rubusoside, Poor solubility, Curcumin, Resveratrol, Cancer

## Abstract

Many anti-cancer drugs have a common problem of poor solubility. Increasing the solubility of the drugs is very important for its clinical applications. In the present study, we revealed that the solubility of insoluble drugs was significantly enhanced by adding rubusoside (RUB). Further, it was demonstrated that RUB could form micelles, which was well characterized by Langmuir monolayer investigation, transmission electron microscopy, atomic-force microscopy, and cryogenic transmission electron microscopy. The RUB micelles were ellipsoid with the horizontal distance of ~25 nm and vertical distance of ~1.2 nm. Insoluble synergistic anti-cancer drugs including curcumin and resveratrol were loaded in RUB to form anti-cancer micelles RUB/CUR + RES. MTT assay showed that RUB/CUR + RES micelles had more significant toxicity on MCF-7 cells compared to RUB/CUR micelles + RUB/RES micelles. More importantly, it was confirmed that RUB could load other two insoluble drugs together for remarkably enhanced anti-cancer effect compared to that of RUB/one drug + RUB/another drug. Overall, we concluded that RUB-based micelles could efficiently load insoluble drugs for enhanced anti-cancer effect.

**Electronic supplementary material:**

The online version of this article (doi:10.1186/s11671-017-2054-4) contains supplementary material, which is available to authorized users.

## Background

Although chemotherapy is one of the most commonly used approaches to treat cancer, conventional chemotherapeutics usually led to numerous unfavorable side effects owing to insolubility, multi-drug resistance, and poor selectivity towards cancer cells [1, 2].

**Fig. 1:**
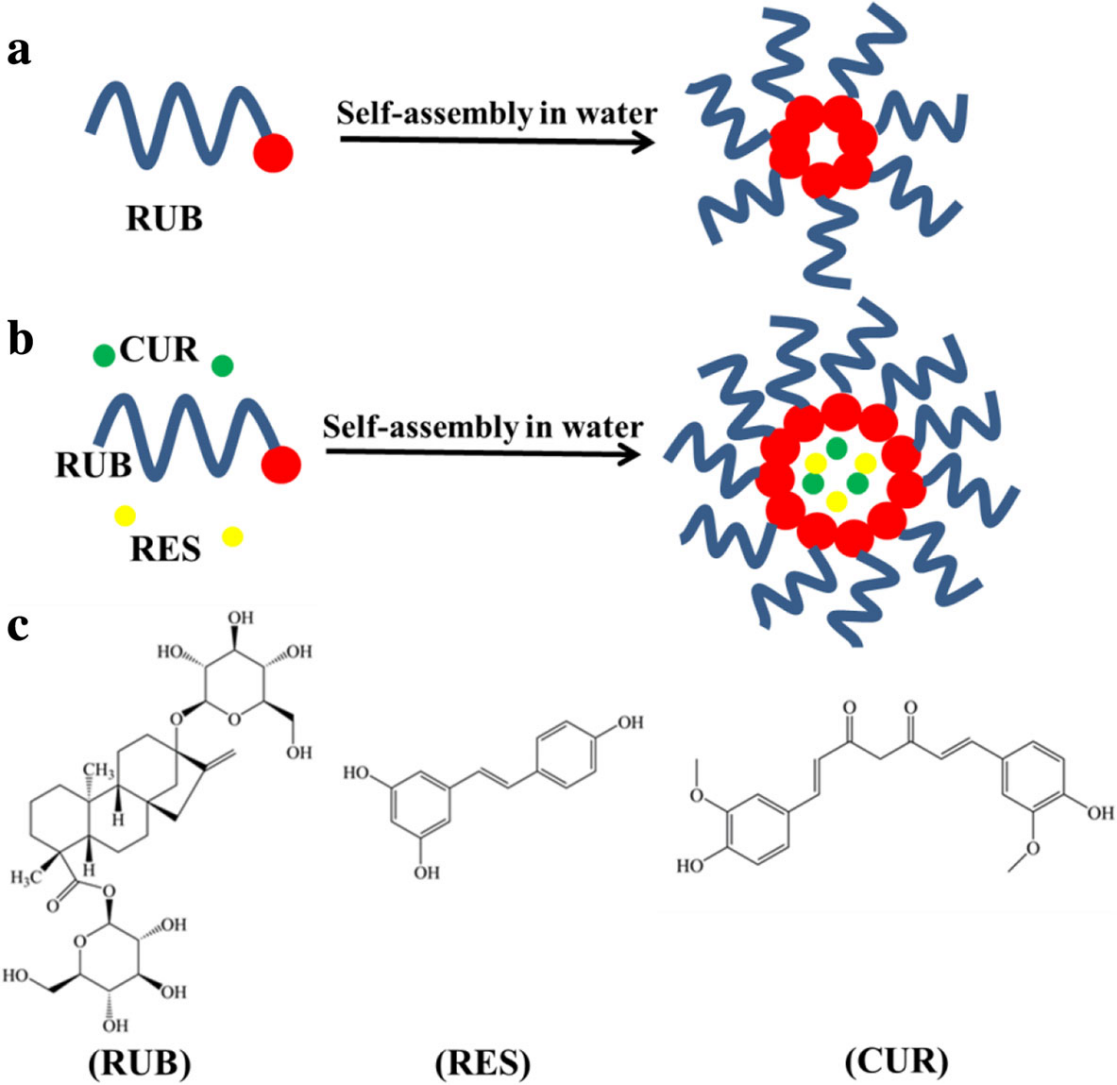
Illustration of the preparation of blank RUB and drug-loaded RUB (RUB/CUR + RES) nanoparticles. **a** The preparation of blank RUB nanoparticles. **b** The preparation of RUB/CUR + RES nanoparticles. **c** Chemical structure of RUB, RES, and CUR

Curcumin (CUR; Fig. 1c) had been exhibited to be a useful inhibitor of the proliferation of several tumor cells [3, 4]. Resveratrol (RES; Fig. 1c), a natural product with low toxicity discovered in grapes and many medical plants, had been extensively investigated as having anti-cancer, anti-inflammatory, and neuroprotective effects [5–9].

Combination therapy usually resulted in survival advantage over monotherapy, which had become a common approach for the treatment of most cancer. Both CUR and RES could act as inducers of chromosomal aberrations leading to cell death or apoptosis in cancer cell lines [10–13]. They could synergistically cause apoptosis in breast cancer cells induced by cigarette smoke [14]. Although the safety and efficacy of CUR and RES synergistically against some diseases had been reported, their further application had been limited owing to poor solubility [15]. So, enhancing their solubility was much essential for their pharmaceutical applications.

Rubusoside (RUB; Fig. 1c) is a diterpene glycoside mainly from Chinese sweet leaf tea leaves (*Rubus suavissimus*; *Rosaceae*) [16]. It was a well-known natural sweetening agent and had been used in food and beverage products. Recently, RUB has been increasingly attracting attention for its solubilizing properties [17–20]. However, the solubilization mechanism of RUB is still unclear until now.

In this study, RUB was used as a solubilizer for CUR and RES solubilization, the solubilization mechanism was investigated with Langmuir monolayer measurement, TEM, cryo-TEM, and AFM. It was demonstrated that RUB could form micelles. Further, the synergistic anti-cancer effects of CUR and RES in different RUB-based micelle formulations were determined on MCF-7 cells.

## Methods

### Materials

Rubusoside, curcumin, and resveratrol were obtained from Shanghai Qiaoyu Company. Dulbecco’s modified Eagle’s medium (DMEM; high glucose), fetal bovine serum (FBS; Australian origin), penicillin and streptomycin, and EDTA solution (0.25% trypsin with 0.53 mM EDTA) were purchased from Life Technologies (Grand Island, NY, USA). MTT reagent was purchased from Sigma-Aldrich (St. Louis, MO, USA). All other chemicals were analyzed by HPLC or of analytical grade.

### High-Performance Liquid Chromatography (HPLC) Measurement

A HPLC system (1260, Agilent, USA) was used for the analyses. All of the analyses were carried out on a Diamonsil ODS C18 HPLC column at 25 °C. For the detection of RUB, elution was performed with acetonitrile and water (*v*/*v*, 33:67). For CUR detection, elution was conducted with methanol and 3.6% acetic acid (*v/v*, 75:25). For RES detection, elution was completed with acetonitrile and 0.5% acetic acid (*v/v*, 54:46). For ginsenoside (Rh_2_) (Additional file 1: Figure S1a) detection, elution was performed with acetonitrile and water (*v/v*, 70:30). For silymarin (SM) (Additional file 1: Figure S1b) detection, elution was set with methanol/acetonitrile/1% acetic acid acetonitrile (*v/v*, 40.4:9.6:50). The flow rate of the mobile phase was 1.0 ml/min and the injection volume of the sample was 20 μl. The selected detection wavelength was 426 nm for CUR, 215 nm for RUB, 306 nm for RES, 203 nm for Rh_2_, and 288 nm for SM. Each sample was subjected to a final step of filtration with a 0.45-μm nylon filter before injection.

### Preparation of RUB-Based Micelles

The appropriate amounts of RUB, CUR, and RES were added into a bottle and vortexed slightly to form a suspension solution. The emulsion was then subjected to an autoclave at 121 °C and 0.11 MPa for 60 min. Samples after heating in the autoclave were supersaturated solutions showing CUR and RES partial precipitation. Then, it was kept in an incubator at 25 °C for 12 h to equilibrate. Finally, each was subjected to a final step of filtration with a 0.45-μm cellulose membrane filter. All products were protected from light and kept at room temperature.

### Langmuir Monolayer Measurement

The RUB formulations were dissolved in chloroform/methanol (*v/v*, 9:1) and be deposited onto the subphase of the minitrough (KSV, Finland) using a microsyringe. Compression was initiated to allow the solvent evaporation. The compression rate was 10 mm/min. Surface pressure-molecular area (π-A) isotherms were determined and processed with the Layer-Builder Analysis Software (KSV). The experiments were performed at 25 °C.

### Characterization of RUB-Based Micelles

Surface morphology of RUB-based micelles was measured by transmission electron microscopy (JEM-2100, 200 kV). Vitrified specimens for cryogenic transmission electron microscopy (Cryo-TEM; Tecnal G20) [21] imaging were in a controlled environment vitrification system (CEVS) at 25 °C and 100% relative humidity. The morphology [22] of the RUB-based micelles was further examined using atomic-force microscopy (AFM; E-Sweep, Seiko, Japan). The sample was prepared by placing a drop onto mica (Asheville-Schoonmaker Mica Co, Newport News, VA). Subsequently, the sample was imaged by scanning 1 μm × 1 μm areas in tapping mode using an OMCL-AC160TS cantilever with 115–190 kHz resonance frequencies and a constant force ranging from 2.5–10 N/m. The size and zeta potential of the preparations were investigated with a Nano ZS90 Zetasizer (Malvern Instruments Ltd., Malvern, UK). The phase transition process of RUB-based micelles was performed using differential scanning calorimetry (NETZSCH Gerätebau GmbH, Selb, Germany) at a heating rate of 10 °C/min from 30 to 250 °C. X-ray diffraction analysis (XRD; D8 Advance, Bruker, Germany) was applied to further investigate the physical state of RUB-based micelles.

### Critical Micelle Concentration (CMC) Measurement

The CMC of RUB micelles were measured by fluorescence measurement using pyrene as a probe [23–25]. The fluorescence emission spectra of pyrene (6 × 10^−7^ M) in different concentrations (varying from 0.01 to 0.4 mM) of RUB solution were determined using a fluorescence spectrophotometer, with the excitation wavelength of 335 nm. The intensities of I3 (394 nm) and I1 (378 nm) were measured at the wavelengths corresponding to the third and first highest energy bands. Then, the intensity ratio of I3 to I1 (I3/I1) in the pyrene emission spectra was calculated.

### In Vitro Drug Release

The drug (CUR and RES) release from RUB/CUR + RES micelles and RUB/RES micelles + RUB/CUR micelles were investigated by a dialysis method in 100 ml of phosphate-buffered saline (PBS; pH 7.4) containing 0.5% Tween 80 at 37 °C [26]. Two milliliters of the sample was placed in a dialysis bag (molecular weight cutoff, 14,000). The bag was then tied and immersed in medium in a shaker bath (100 strokes/min). At a defined time interval, 100 μl of the sample was withdrawn and replaced with the same volume of fresh medium. The drug concentration was measured by HPLC.

### Cell Culture

MCF-7 cells were purchased from the Cell Bank of Chinese Academy of Sciences (Shanghai, China). They were cultured at 37 °C under 5% CO_2_ in DMEM supplemented with 10% fetal bovine serum (PAA, Austria). The cultured cells were trypsinized once 80% confluence with 0.25% trypsin-EDTA solution (Sigma, USA).

### Cell Apoptosis Measurement

Annexin V-FITC/PI dual staining for apoptosis was performed to measure the apoptosis. Briefly, 1 × 10^5^ cells/well of MCF-7 cells were seeded in a six-well cell culture plate and incubated for 24 h. Subsequently, cells were exposed to free RES + CUR, RUB/CUR + RES micelles, or RUB/RES micelles + RUB/CUR micelles. Cells were harvested post-incubation by trypsinization and washed twice with PBS. Then, cells were resuspended in binding buffer and stained with Annexin V-FITC and PI detection kit according to the protocol provided by the manufacturer. Stained cells were analyzed by a FACS Aria II flow cytometer.

### Anti-Cancer Effect of RUB-Based Micelles

The anti-cancer effect of RUB-based micelles on MCF-7 cells was assessed via MTT method. Cells (1 × 10^5^ cells/ml) were cultured overnight in 96-well plates. Subsequently, they were treated with RES + CUR, RUB/CUR + RES micelles or RUB/RES micelles + RUB/CUR micelles at the concentration of CUR (23 μM) and RES (110 μM) for 24 h. Control cells were treated with PBS. At indicated time points, 10 μl MTT (5 mg/ml) was added and plates were incubated at room temperature for another 4 h in the dark. Then, the medium was replaced with 150 μl DMSO, and plates were further incubated for 10 min. OD_570nm_ was measured using a microplate reader (Bio-Rad Laboratories Inc, Hercules, CA, USA).

### Cellular Localization of RUB-Based Micelles in MCF-7 Cells

To image the intracellular localization of the micelles, MCF-7 cells were incubated with free coumarin-6 (C6) or C6-loaded micelles (C6 content, 4.5 mg/ml) for 15 min, 1 h, and 2 h at 37 °C. After that, the culture medium was aspirated and cells were washed three times with PBS, followed by cell fixation with 4% paraformaldehyde. Next, the nucleus was stained with DAPI for 10 min at 37 °C. The fluorescence was then visualized using a confocal laser scanning microscope (LSM710, Zeiss, Germany).

### Cellular Uptake by Flow Cytometry

MCF-7 cells were seeded on six-well culture plates (1 × 10^5^ cells/well) and incubated for 24 h in DMEM medium containing 10% FBS. Subsequently, they were treated with free coumarin-6 (C6) or C6-loaded micelles (with an equivalent C6 concentration of 4.5 mg/ml). After incubation, they were harvested and suspended in 500 μl of PBS. The cellular uptake of micelles was determined using a flow cytometry (Becton Dickinson, FACS, Aria II).

### Statistical Analysis

All data were represented as mean ± SD from at least three independent experiments. Statistical significance analysis was conducted using Student’s *t* test. *P* < 0.05 was considered statistically significant.

## Results and Discussion

### Characterization of the RUB-Based Nanoparticles

**Fig. 2:**
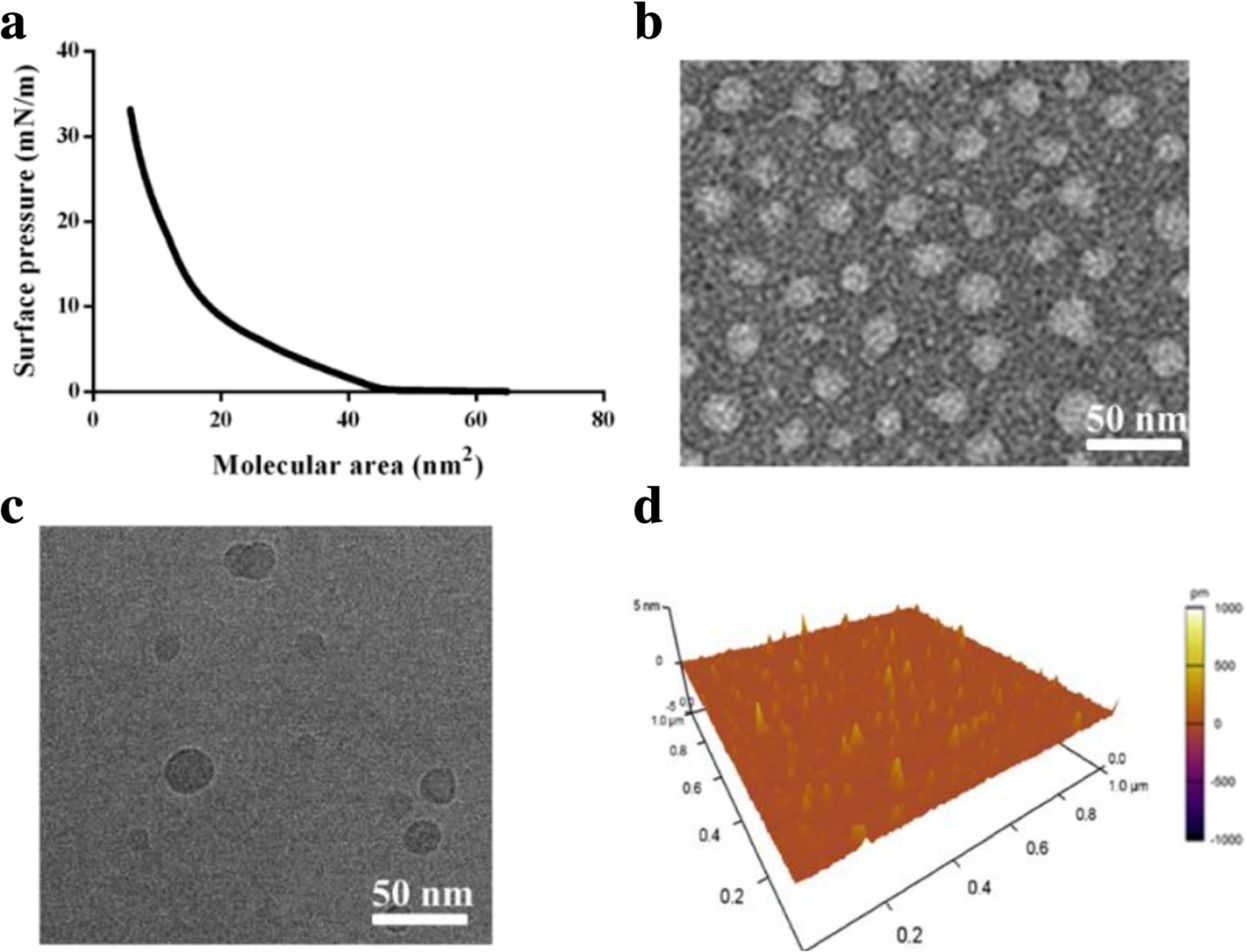
Characterization of blank RUB nanoparticles. **a** The surface pressure-molecular area isotherms of RUB nanoparticles. **b** Transmission electron microscopy (TEM) image of RUB nanoparticles. **c** The corresponding cryogenic transmission electron microscopy (Cryo-TEM) image of RUB nanoparticles. **d** Atomic-force microscopy (AFM) image of RUB nanoparticles

The behavior of Langmuir monolayers at interfaces could reveal the amphiphilic and self-assembled properties of amphiphiles [27, 28]. The different π-A isotherms of blank RUB particles showed that RUB molecular area was 25 nm^2^ and the collapse pressure of the RUB Langmuir monolayer was merely 33 mN/m (Fig. 2a). It indicated that RUB had amphipathic features, which could be beneficial for micelle formation.

As outlined in Fig. 1b, c, RUB and RUB/CUR + RES could form self-assembled nanoparticles in aqueous condition. It was proved that RUB could form round nanoparticles of about 25 nm in diameter by TEM (Fig. 2b). Cryo-TEM result indicated that the morphology of nanoparticles was round with hollow (Fig. 2c) and its measured size is also ~25 nm. AFM measurement further verified that RUB-formed nanoparticles were ellipsoid with the horizontal distance of ~25 nm and vertical distance of ~1.2 nm (Fig. 2d and Additional file 1: Figure S2).

### The Critical Micelle Concentrations (CMC) of RUB Micelles

**Fig. 3:**
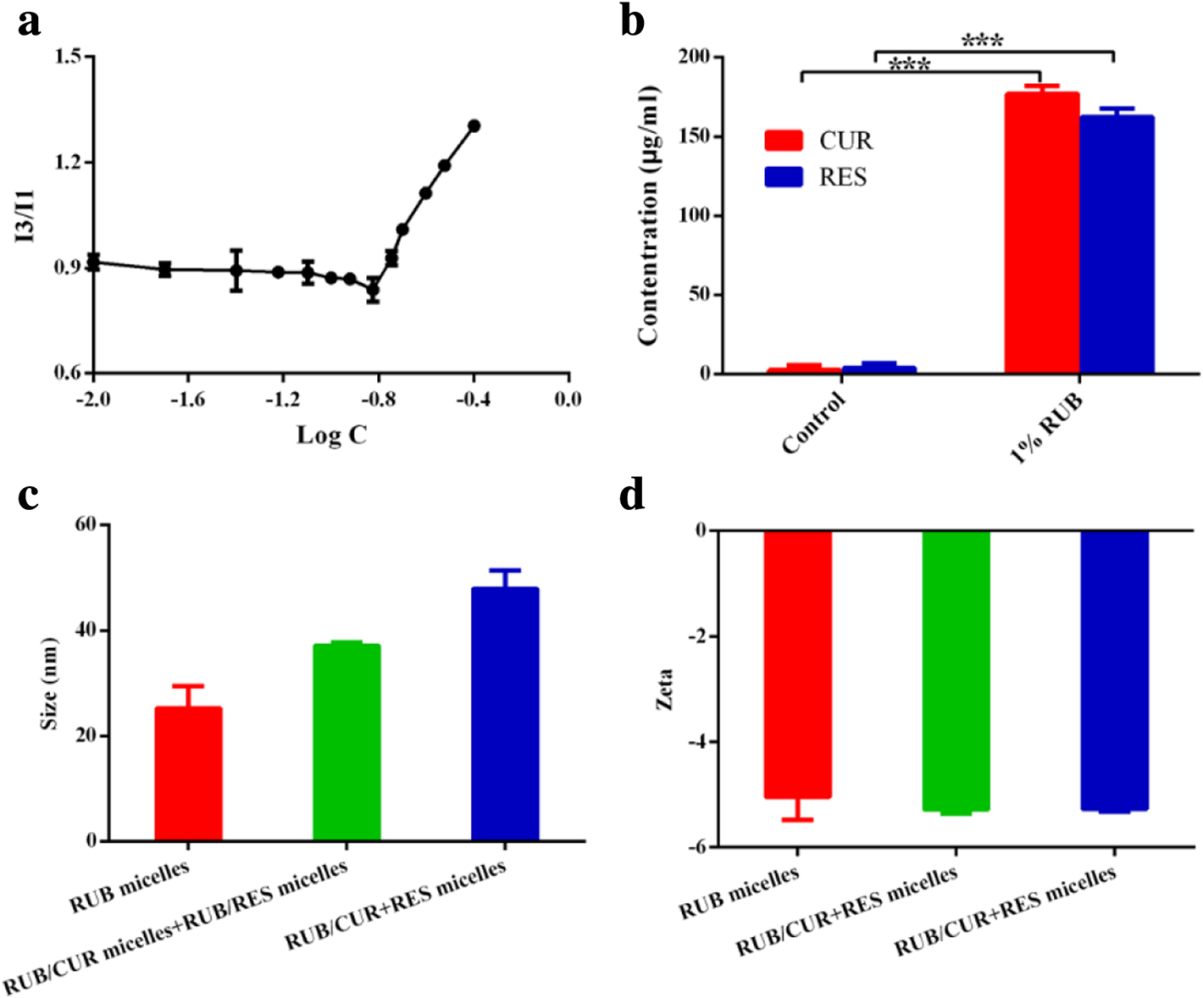
Characterization of RUB-based micelles. **a** Determination of critical micelle concentrations (CMC) of RUB micelles using pyrene as fluorescent probe. The CMC was calculated as 0.18 mM. **b** RUB micelles were adopted to load CUR and RES for significantly enhancing their solubility. ***P < 0.001 (two-tailed Student’s *t* test). **c**, **d** The size and zeta potential of RUB-based micelles were measured by dynamic light scattering (DLS). *Error bars* indicated s.d. (*n* = 3)

When in the lower molecular concentration, the value of I3/I1 remained nearly constant. Once the molecular concentration reached above the CMC, the value of I3/I1 increased significantly, indicating the formation of micelles [29]. The CMC value can reveal the self-aggregation ability of the amphiphilic molecules. It showed the change in the value of I3/I1 against the logarithm of RUB micelle concentration (Fig. 3a). The CMC value of blank RUB micelles was determined to be 0.18 mM in deionized water. The insoluble anti-cancer drugs CUR and RES were encapsulated into RUB micelles to form anti-cancer micelles RUB/CUR + RES. As shown in Fig. 3b, robust solubility increases by 60-fold of CUR and 33-fold of RES were detected in RUB/CUR + RES micelles. It indicated that RUB micelles were an excellent insoluble drug carrier.

### Size and Zeta Potential of RUB-Based Micelles

The particle size and surface zeta potential of RUB micelles, RUB/CUR + RES micelles, and RUB/CUR micelles + RUB/RES micelles are shown in Fig. 3c, d, which was measured by DLS. Micelles with smaller size tended to accumulate easily in tumor sites due to the enhanced permeability and retention (EPR) effect and gained a faster internalization rate into cells [30, 31]. Results showed that the sizes of RUB-based micelles were all small micelles in different ways (Fig. 3c), which might benefit from well hydrophobic interaction between the hydrophobic cores of RUB micelles and insoluble drugs. The three kinds of RUB-based micelles had a similar zeta potential value because of their similar surface characterization (Fig. 3d).

### X-Ray Diffraction (XRD) Measurement

**Fig. 4:**
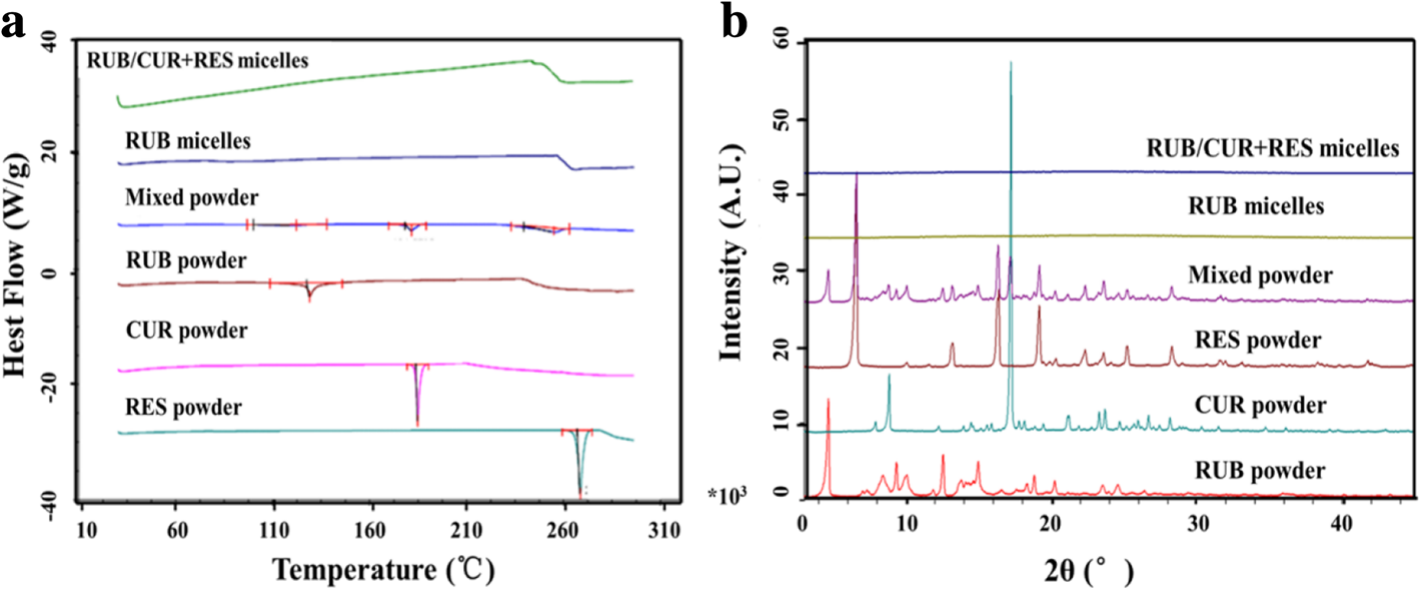
Characterization of RUB/CUR + RES micelles. **a** X-ray diffraction of RUB/CUR + RES micelles. **b** Differential scanning calorimetry of RUB/CUR + RES micelles

XRD was used to evaluate the crystallization behavior of raw materials and RUB-based micelles. As shown in Fig. 4a, the raw materials showed the presence of different peaks. The intensity of the peaks for RUB-based micelles disappeared compared to raw materials in the diffractogram, which showed disappeared crystallinity of the drugs in the micelles.

### Differential Scanning Calorimeter (DSC) Analysis

DSC measurements were used to acquire information on the crystallinity and polymorphism of the interaction between the drug and micelles from DSC thermograms. It could provide information including the appearance of new peaks, the elimination of endothermic peaks, and changes in peak shape and onset, peak temperature, or enthalpy [32]. As shown in Fig. 4b, the thermograms of the physical mixture of raw materials showed three peaks at 131.9, 179.2, and 266.1 °C, which well corresponded to RUB power’s peak at 135.6 °C, CUR power’s peak at 181.2 °C, and RES power’s peak at 269.8 °C. The peaks of RES power and CUR power disappeared when they were loaded in RUB micelles to form RUB/CUR + RES micelles, which indicated that raw materials lost its crystallinity [33, 34].

### Cell Uptake of RUB-Based Micelles in MCF-7 Cells

**Fig. 5:**
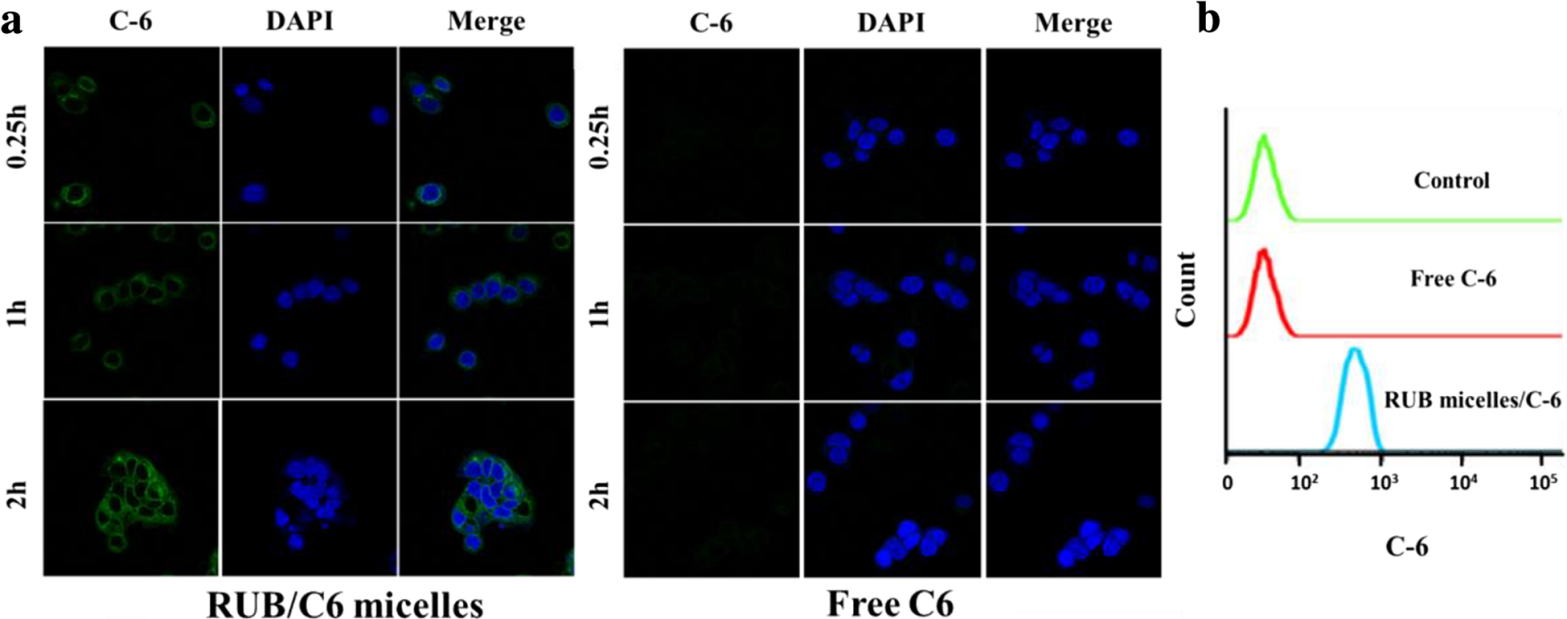
In vitro uptake behavior of RUB-based micelles in breast cancer cell model MCF-7. **a** Fluorescence micrographs of MCF-7 cells were imaged after incubation with free C6 or RUB/C6 micelles for 2 h (excitation 488 nm, emission 500~530 nm). *Scale bars* 10 μm. **b** Cell binding of indicated C6 formulations (4.5 mg/ml) on MCF-7 cells at 2 h was measured by flow cytometry (FACS)

Coumarin-6 (C6), a fluorescence marker, had been widely used as a probe to substitute hydrophobic drugs in cell uptake experiments owing to its high fluorescence activity and biocompatibility. Herein, free C6 and C6-loaded micelles were incubated with the abovementioned MCF-7 cells; the cell nuclei were stained with DAPI and then imaged with a confocal laser scanning microscope. As shown in Fig. 5a, the cell nuclei were surrounded by green fluorescence, indicating that C6-loaded micelles had been internalized into cells. However, the free C6 group exhibited nearly none green fluorescence intensity compared to the C6-loaded micelles group. Furthermore, flow cytometric analysis demonstrated a similar intracellular uptake pattern corresponding to the confocal data at 2 h (Fig. 5b). Further, we investigated the uptake behavior of free C6 and C6-loaded micelles in Caco-2 cells. The free C6 group also exhibited nearly none green fluorescence intensity compared to C6-loaded micelles group (Additional file 1: Figure S3). These results suggest that the RUB micelles played an important role in enhancing intracellular uptake of hydrophobic drugs.

### In Vitro Anti-Cancer Effect of RUB-Based Micelles

Here, cytotoxicity of blank RUB micelles on MCF-7 cells was evaluated. It demonstrated that high cell viabilities in blank RUB micelles at a concentration of 4–32 mmol/l and declined cell viabilities (at a concentration of above 32 mmol/l) in MCF-7 cells (Additional file 1: Figure S4), indicating that blank RUB micelles does not bring significant additional toxicity to cells at a concentration of below 32 mmol/l. This result confirmed blank RUB micelles are biocompatible.

**Fig. 6:**
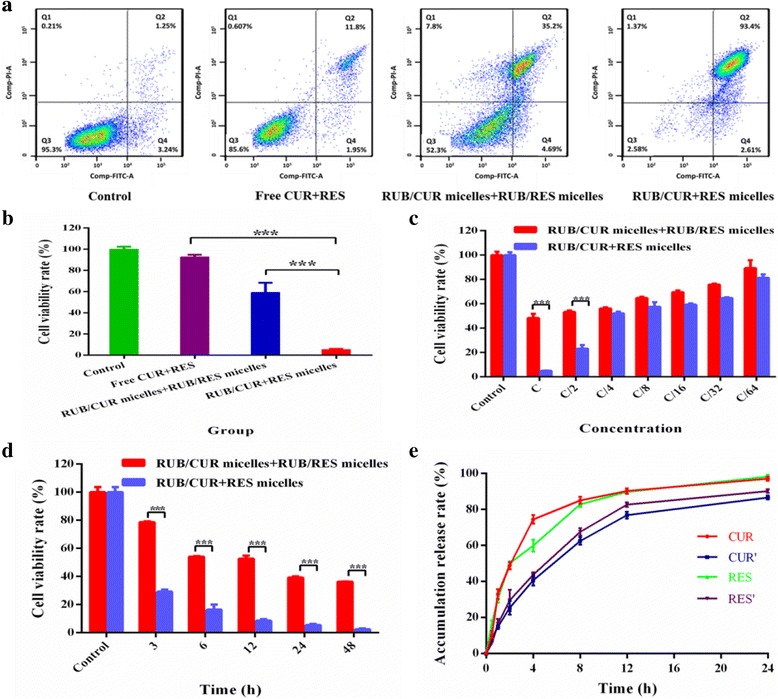
The anti-cancer effects of RUB-based micelles on MCF-7 cells. **a** Apoptosis of MCF-7 cells treated with indicated formulations of CUR (23 μM) and RES (110 μM) for 24 h was analyzed by flow cytometry using Annexin V-FITC/PI staining. **b** Cell viability of MCF-7 cells was measured by MTT method, which was treated with indicated formulations of CUR (23 μM) and RES (110 μM) for 24 h. **c**, **d** Time- and dose-dependent manners of cell viability of MCF-7 cells treated with indicated formulations were measured by MTT method. **c** represented for CUR (23 μM). RES’ concentration was 110 μM. **e** In vitro release profile of CUR and RES from RUB/CUR + RES micelles and RUB/CUR micelles + RUB/RES micelles in PBS buffer (pH 7.4). *Error bars* indicated s.d. (*n* = 3). ***P < 0.001 (two-tailed Student’s t test)

We investigated the anti-cancer potential of the combination treatment with different formulations on MCF-7 cells. As presented in Fig. 6a, the percentage of the latest apoptosis of free CUR + RES, RUB/CUR + RES micelles, and RUB/CUR micelles + RUB/RES micelles in MCF-7 cells was 11.8, 93.4, and 35.2%, respectively. These results demonstrated that free drug (CUR, RES) in combination caused slight cytotoxicity on MCF-7 cells; it was further improved by RUB micelles’ encapsulation. More strikingly, the cytotoxicity of RUB/CUR + RES was significantly higher than that of RUB/CUR + RUB/RES, which indicated two drugs were encapsulated in micelles together which had much better anti-cancer effect compared to in micelles, respectively. In order to further confirm this, MTT assay was conducted to reveal that the cytotoxicity of RUB/CUR + RES on MCF-7 cells was significantly higher than that of RUB/CUR + RUB/RES (Fig. 6b). Simultaneously, this trend presented in a time- and dose-dependent manner (Fig. 6c, d). Finally, the cytotoxicity mechanism was investigated by drug accumulation release rate in PBS (pH 7.4). At 8 h, it showed that the release rate of CUR and RES was 85 and 82% in RUB/CUR + RES micelles, which was higher than that of CUR (62%) and RES (67%) in RUB/CUR micelles + RUB/RES micelles (Fig. 6e), which contributed to the high cytotoxicity of RUB/CUR + RES micelles. The pH value of tumor microenvironment is acid, so drug accumulation release rate in PBS (pH 5.5) was further conducted (Additional file 1: Figure S5). At 0.5 h, in RUB/CUR + RES micelles, the release rate of CUR (19.03%) and RES (26.8%) in PBS (pH 5.5) was faster than that of CUR (10.60%) and RES (16.70%) in PBS (pH 7.4). The similar drug release trends in PBS (different pH value) were found in RUB/CUR micelles + RUB/RES micelles formulation, which was benefit for tumor therapy.

Based on our results that RUB micelles loaded two insoluble drugs together had remarkably enhanced anti-cancer effect than that of RUB/one drug + RUB/another drug, it is highly interesting to explore the possibility in extending RUB-based micelles to other two insoluble drug encapsulation. Previous reports have revealed that ginsenoside (Rh2) and silymarin (SM) had therapeutic effects for some types of cancer and were insoluble [35, 36].

**Fig. 7:**
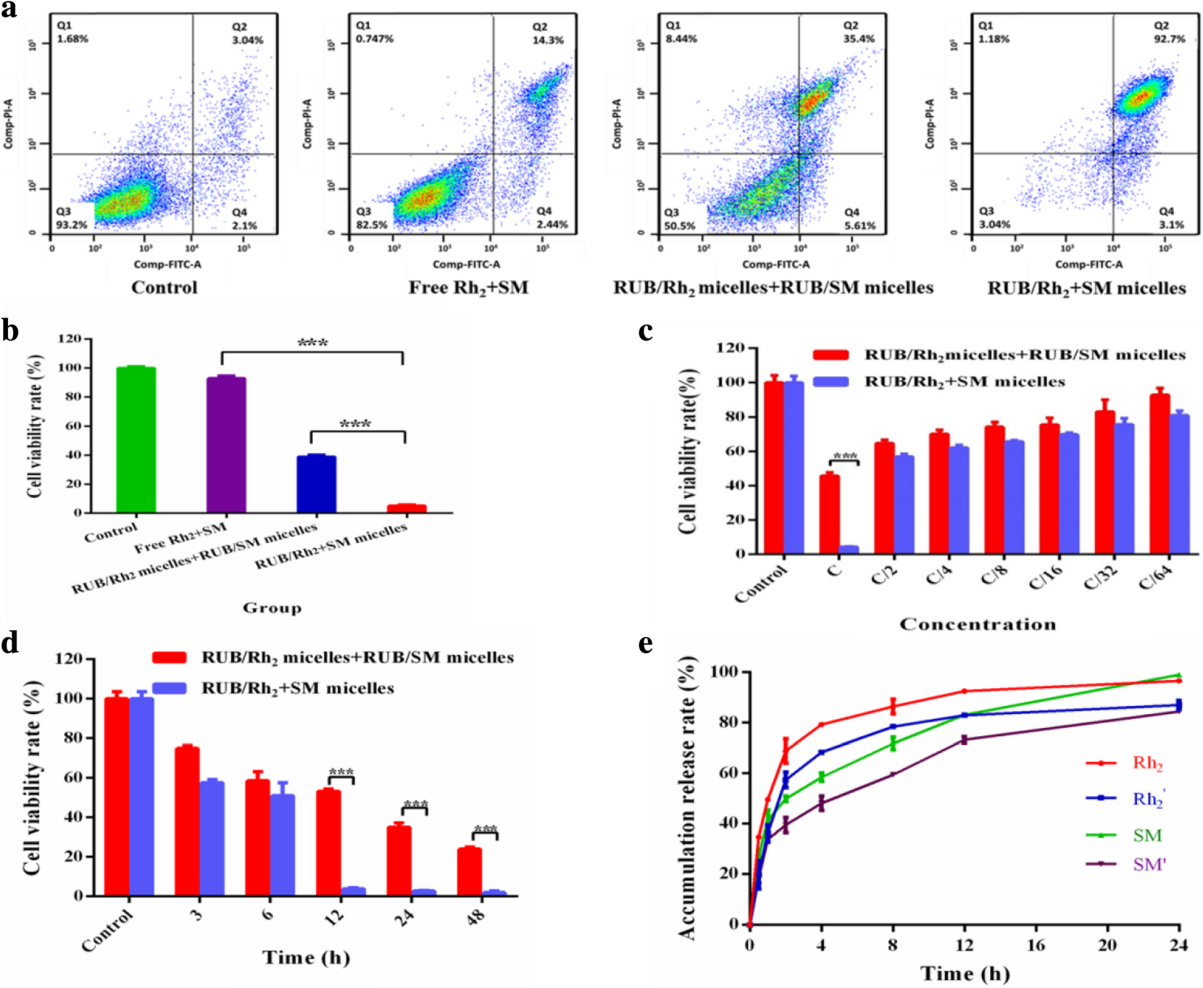
The anti-cancer effects of RUB-based micelles on MCF-7 cells. **a** Apoptosis of MCF-7 cells treated with indicated formulations of Rh2 (2.19 mM) and SM (0.26 mM) for 24 h was analyzed by flow cytometry using Annexin V-FITC/PI staining. **b** Cell viability of MCF-7 cells was measured by MTT method, which was treated with indicated formulations of Rh2 (2.19 mM) and SM (0.26 mM) for 24 h. **c**, **d** Time- and dose-dependent manners of cell viability of MCF-7 cells treated with indicated formulations were measured by MTT method. **c** represented for Rh2 (2.19 mM). SM’s concentration was 0.26 mM. **e** In vitro release profile of Rh2 and SM from RUB/Rh2 + SM micelles and RUB/Rh2 micelles + RUB/SM micelles. *Error bars* indicated s.d. (*n* = 3). ***P < 0.001 (two-tailed Student’s *t* test)

Herein, we have investigated the anti-cancer potential of the combination treatment with different formulations on MCF-7 cells using drug SM and Rh2. As demonstrated in Fig. 7a, free drug SM and Rh_2_ in combination caused slight cytotoxicity on MCF-7 cells; the cytotoxicity was further improved by RUB micelles’ encapsulation. More critically, the cytotoxicity of RUB/Rh_2_ + SM micelles was significantly higher compared to RUB/Rh_2_ micelles + RUB/SM micelles, indicating the two insoluble drugs encapsulated in micelles together had much better anti-cancer effect than in micelles, respectively. Further, MTT assay revealed that the cytotoxicity of RUB/Rh_2_ + SM micelles on MCF-7 cells was significantly higher compared to that of RUB/Rh_2_ micelles + RUB/SM micelles (Fig. 7b). This trend also had a time- and dose-dependent manner (Fig. 7c, d). The mechanism result revealed that the release rate of Rh_2_ and SM was 86 and 72% in RUB/Rh_2_ + SM micelles (PBS, pH 7.4), which was higher than that of Rh_2_ (78%) and SM (59%) in RUB/Rh_2_ micelles + RUB/SM micelles (Fig. 7e). Drug accumulation release rate in PBS (pH 5.5) was further investigated (Additional file 1: Figure S6). The release rate of Rh2 and SM in PBS (pH 5.5) was also faster than that in PBS (pH 7.4).

## Conclusions

In this study, it was proved that RUB was self-assembled to form micelles. The RUB-based micelle system developed in this study was a promising small molecule carrier that efficiently improved the solubility of insoluble drugs. CUR and RES were loaded in RUB to form anti-cancer micelles RUB/CUR + RES. Interestingly, RUB/CUR + RES micelles had more remarkable toxicity on MCF-7 cells compared to RUB/CUR micelles + RUB/RES micelles. More importantly, it was proved that RUB could load other two insoluble drugs together for remarkably enhanced anti-cancer effect compared to that of RUB/one drug + RUB/another drug. Overall, RUB-based micelles could efficiently load insoluble anti-cancer drugs for significantly enhanced anti-cancer effect.

## Additional file

Additional file 1: Figure S1. The structure of ginsenoside (a) and silymarin (b). **Figure S2.** Topograph AFM images (a) and height profile (b) of RUB-based nanoparticles. **Figure S3.** Cell uptake of free C6 and RUB/C6 micelles in Caco-2 cells was imaged by confocal laser scanning microscope. Scale bars 10 μm. **Figure S4.** Cell viability of MCF-7 cells treated with RUB micelles. **Figure S5.** In vitro release profile of CUR and RES from RUB/CUR + RES micelles and RUB/CUR micelles + RUB/RES micelles in PBS buffer (pH 5.5). **Figure S6.** In vitro release profile of Rh2 and SM from RUB/Rh2 + SM micelles and RUB/Rh2 micelles + RUB/SM micelles in PBS buffer (pH 5.5). (DOCX 406 kb)
