# Supplementary material for: A Novel Solubility-Enhanced Rubusoside-Based Micelles for Increased Cancer Therapy
Source: Nanoscale Res Lett. 2017 Apr 13;12:274. doi: 10.1186/s11671-017-2054-4 (PMC5391341; doi:10.1186/s11671-017-2054-4)
Supplement: Additional file 1: Figure S1. — The structure of ginsenoside (a) and silymarin (b). Figure S2. Topograph AFM images (a) and height profile (b) of RUB-based nanoparticles. Figure S3. Cell uptake of free C6 and RUB/C6 micelles in Caco-2 cells was imaged by confocal laser scanning microscope. Scale bars 10 μm. Figure S4. Cell viability of MCF-7 cells treated with RUB micelles. Figure S5. In vitro release profile of CUR and RES from RUB/CUR + RES micelles and RUB/CUR micelles + RUB/RES micelles in PBS buffer (pH 5.5). Figure S6. In vitro release profile of Rh2 and SM from RUB/Rh2 + SM micelles and RUB/Rh2 micelles + RUB/SM micelles in PBS buffer (pH 5.5). (DOCX 406 kb) [file 11671_2017_2054_MOESM1_ESM.docx]

Supplementary materials:


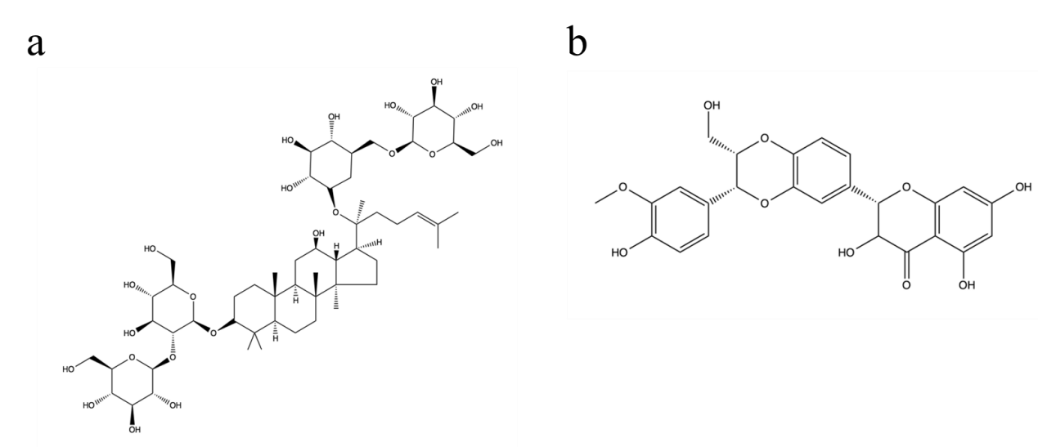


**Fig. S1** The structure of ginsenoside (a)and silymarin (b).


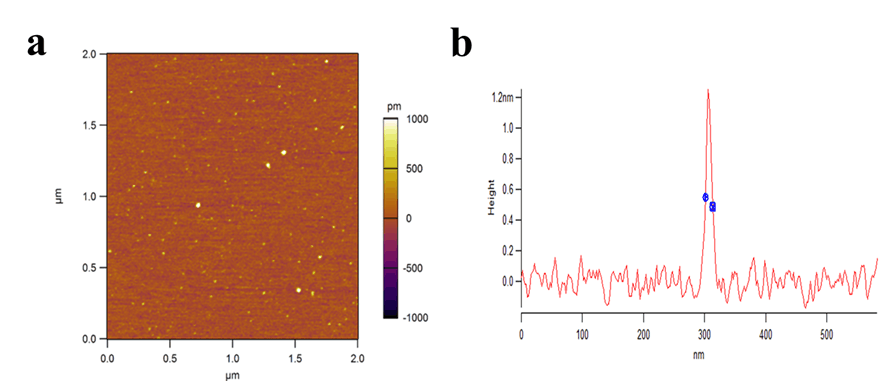


**Fig. S2** Topograph AFM images(a) and height profile (b) of RUB based nanoparticles.


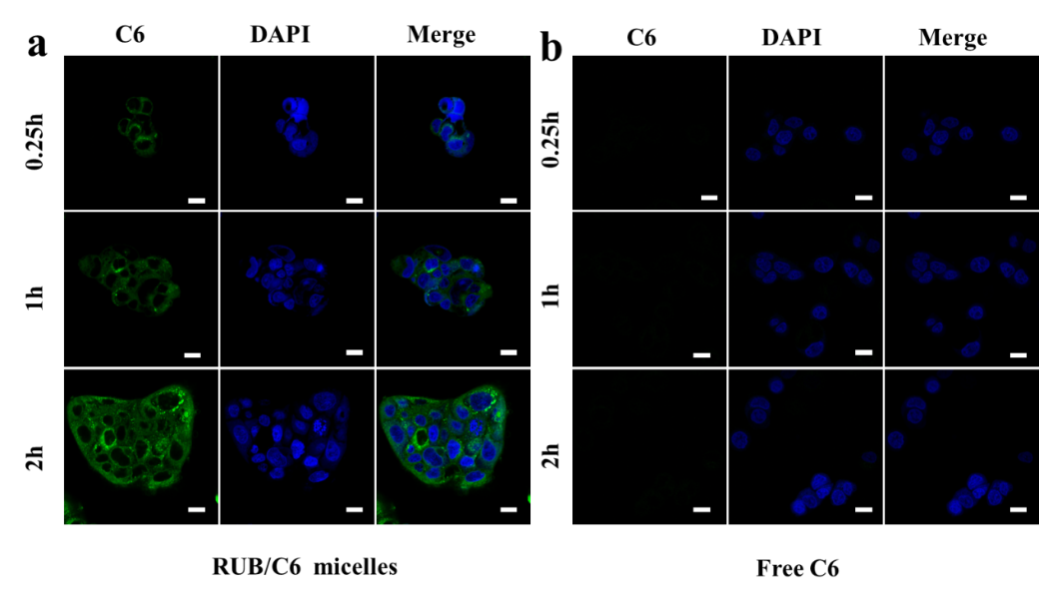


**Fig. S3** Cell uptake of free C6 and RUB/C6 micelles in Caco-2 cells was imaged by confocal laser scanning microscope. Scale bars: 10 μm.





**Fig. S4** Cell viability of MCF-7 cells treated with RUB micelles.


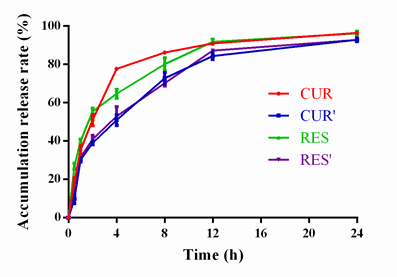


**Fig. S5** In vitro release profile of CUR and RES from RUB/CUR+RES micelles and RUB/CUR micelles+RUB/RES micelles in PBS buffer (pH 5.5).


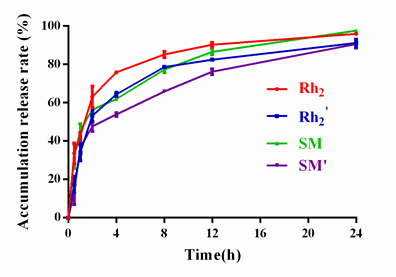


**Fig. S6** In vitro release profile of Rh2 and SM from RUB/Rh2+SM micelles and RUB/Rh2 micelles+RUB/SM micelles in PBS buffer (pH 5.5).
